# A rare case of severe acute pancreatitis complicated by gastric fistula and total splenic liquefaction

**DOI:** 10.1055/a-2626-3869

**Published:** 2025-07-25

**Authors:** Xin Huang, Ximei Cao, Yao Wu, Liang Xia, Yin Zhu, Nonghua Lu, Wenhua He

**Affiliations:** 1 117970 Department of Gastroenterology, Digestive Disease Hospital, The First Affiliated Hospital of Jiangxi Medical College, Nanchang, China; 2 Department of Gastroenterology, Jiujiang City Key Laboratory of Cell Therapy, Jiujiang No. 1 Peopleʼs Hospital, Jiujiang, China

A 44-year-old man was admitted in June 2024 with persistent abdominal pain and a history of diabetes with acute pancreatitis 7 months prior. Laboratory tests showed a white blood cell count of 25.13 × 10⁹/L (neutrophils 92.5%), triglycerides 6.58 mmol/L, and amylase 602.4 U/L. Abdominal CT demonstrated acute pancreatitis with a 4-cm pseudocyst at the pancreatic tail ( Fig. 1 ). During hospitalization, the patient developed recurrent fever, abdominal rigidity, and left upper quadrant tenderness. CT performed at 1 week after disease onset revealed necrotizing pancreatitis with complete absence of the spleen, which was replaced by patchy hypodense areas with gas bubbles. Gastric fistula was identified by a discontinuity in the greater curvature of the upper gastric body ( Fig. 2 ). Despite antibiotics and nutritional support, percutaneous drainage of splenic necrosis and pancreatic walled-off necrosis were necessary ( Fig. 3 ). A nasojejunal tube was endoscopically placed distal to the gastric fistula for enteral nutrition. At week 6, endoscopic necrosectomy via the percutaneous sinus tract was attempted but failed due to large necrotic debris. Successful debridement was achieved endoscopically through the gastric fistula ( Video 1 ). Postoperative CT showed resolution of necrosis, and gastroscopy demonstrated fistula shrinkage ( Fig. 4 ). The patient was discharged clinically improved. Two-month follow-up confirmed complete resolution of peripancreatic necrosis and fistula healing ( Fig. 5 ).

**Fig. 1:**
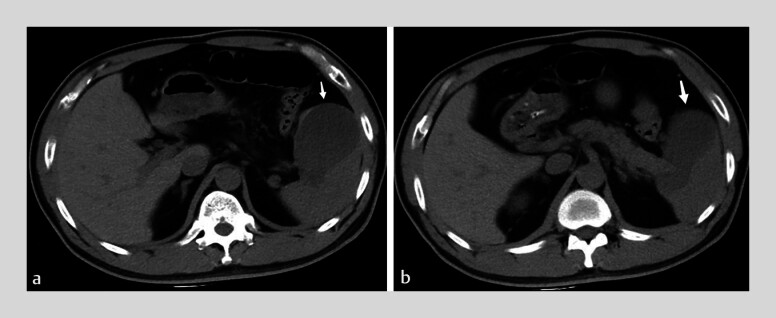
CT demonstrates a pseudocyst at the pancreatic tail adjacent to the spleen (arrows) in a 44-year-old man.

**Fig. 2:**
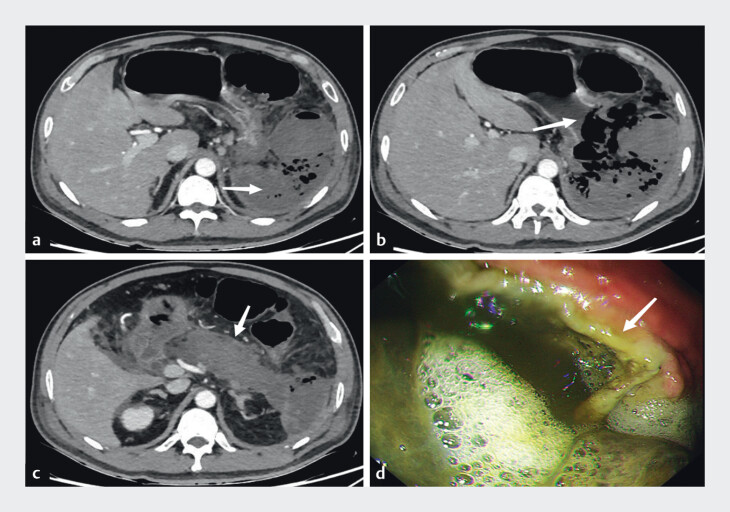
**a** Complete splenic necrosis with absence of splenic parenchyma (arrow). **b** Discontinuity of gastric wall indicating fistula formation (arrow). **c** Global pancreatic necrosis with predominant parenchymal involvement (arrow). **d** Endoscopic view of gastric fistula (arrow).

**Fig. 3:**
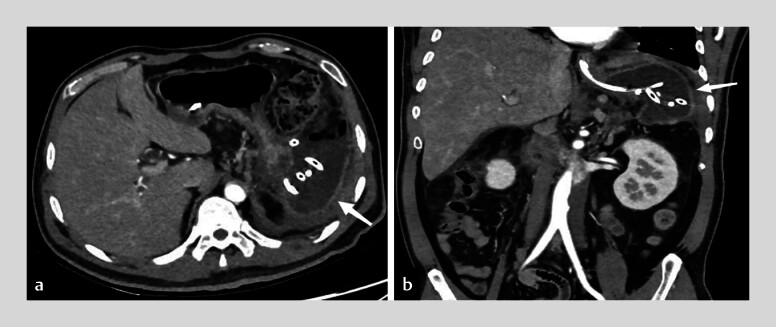
Percutaneous drainage of pancreatic necrosis (arrows indicate catheter).

Endoscopic necrosectomy procedure for pancreatic necrosis.Video 1

**Fig. 4:**
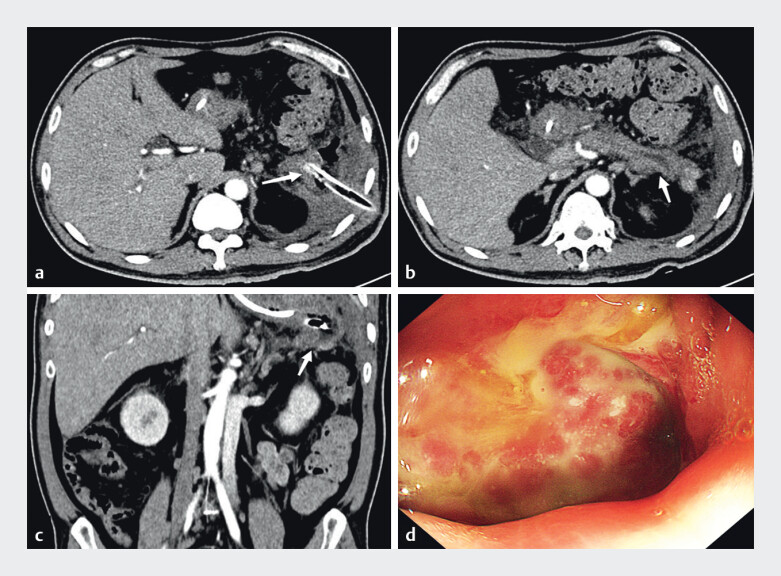
**a–c** Near-complete resolution of necrotic collections after debridement (arrows mark residual cavities). **d** Endoscopic image showing improvement of gastric fistula following necrosectomy.

**Fig. 5:**
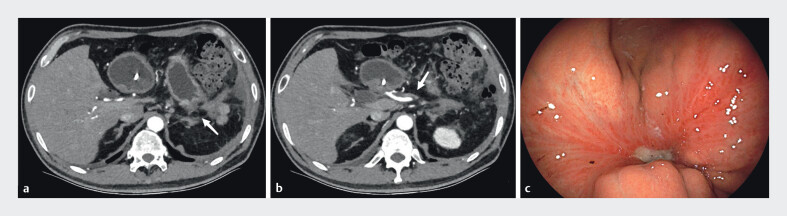
**a, b** Follow-up CT at 2 months shows complete resolution of necrosis (arrows denote original sites). **c** Endoscopic confirmation of near-total fistula healing.

This is the first reported case of severe acute pancreatitis with concurrent gastric fistula and total splenic autolysis. Gastrointestinal fistulas in pancreatitis typically involve the duodenum or colon 1 . Gastric fistulas are rare; the one in this case likely resulted from pancreatic enzyme extravasation and local inflammation. Although splenic involvement can occur in severe acute pancreatitis 2 , complete splenic liquefaction is exceedingly rare. It may be attributed to: (1) direct enzymatic autodigestion by trypsin from the ruptured pancreatic pseudocyst (formed during the prior pancreatitis episode); and (2) splenic vein thrombosis causing ischemic necrosis 3 4 .

Endoscopy_UCTN_Code_CCL_1AZ_2AF

## Notes

Endoscopy E-Videos https://eref.thieme.de/e-videos
*E-Videos* is an open access online section of the journal *Endoscopy* , reporting on interesting cases and new techniques in gastroenterological endoscopy. All papers include a high-quality video and are published with a Creative Commons CC-BY license. Endoscopy E-Videos qualify for HINARI discounts and waivers and eligibility is automatically checked during the submission process. We grant 100% waivers to articles whose corresponding authors are based in Group A countries and 50% waivers to those who are based in Group B countries as classified by Research4Life (see: https://www.research4life.org/access/eligibility/ ). This section has its own submission website at https://mc.manuscriptcentral.com/e-videos .
